# Potent activity of the Hsp90 inhibitor ganetespib in prostate cancer cells irrespective of androgen receptor status or variant receptor expression

**DOI:** 10.3892/ijo.2012.1698

**Published:** 2012-11-14

**Authors:** SUQIN HE, CHAOHUA ZHANG, AYESHA A. SHAFI, MANUEL SEQUEIRA, JAIME ACQUAVIVA, JULIE C. FRIEDLAND, JIM SANG, DONALD L. SMITH, NANCY L. WEIGEL, YUMIKO WADA, DAVID A. PROIA

**Affiliations:** 1 Synta Pharmaceuticals Corp., Lexington, MA 02421;; 2 Department of Molecular and Cellular Biology, Baylor College of Medicine, Houston, TX 77030, USA

**Keywords:** Hsp90 inhibition, ganetespib, androgen receptor, prostate cancer, cancer therapy

## Abstract

Androgen ablation therapy represents the first line of therapeutic intervention in men with advanced or recurrent prostate tumors. However, the incomplete efficacy and lack of durable response to this clinical strategy highlights an urgent need for alternative treatment options to improve patient outcomes. Targeting the molecular chaperone heat shock protein 90 (Hsp90) represents a potential avenue for therapeutic intervention as its inhibition results in the coordinate blockade of multiple oncogenic signaling pathways in cancer cells. Moreover, Hsp90 is essential for the stability and function of numerous client proteins, a number of which have been causally implicated in the pathogenesis of prostate cancer, including the androgen receptor (AR). Here, we examined the preclinical activity of ganetespib, a small molecule inhibitor of Hsp90, in a panel of prostate cancer cell lines. Ganetespib potently decreased viability in all lines, irrespective of their androgen sensitivity or receptor status, and more effectively than the ansamycin inhibitor 17-allylamino-17-demethoxygeldanamycin (17-AAG). Interestingly, while ganetespib exposure decreased AR expression and activation, the constitutively active V7 truncated isoform of the receptor was unaffected by Hsp90 inhibition. Mechanistically, ganetespib exerted concomitant effects on mitogenic and survival pathways, as well as direct modulation of cell cycle regulators, to induce growth arrest and apoptosis. Further, ganetespib displayed robust antitumor efficacy in both AR-negative and positive xenografts, including those derived from the 22Rv1 prostate cancer cell line that co-expresses full-length and variant receptors. Together these data suggest that further investigation of ganetespib as a new therapeutic treatment for prostate cancer patients is warranted.

## Introduction

Prostate cancer is the second leading cause of male cancer-related mortality in the United States (1). A distinctive characteristic of this cancer type is that prostate tumors are critically dependent on androgen for development, growth and survival (2,3). Androgen ablation therapy is the foundation of current prostate cancer treatment for patients that present with locally advanced or metastatic disease. This is typically achieved through chemical castration using selective agents that reduce levels of circulating androgens, such as luteinizing hormone-releasing hormone (LHRH) agonists or androgen receptor (AR) antagonists such as bicalutamide (4). Although this approach initially induces clinical remissions, most patients ultimately relapse and progress to castration-resistant disease within a median of 18–24 months (5).

It is now clear that these advanced tumors continue to rely on AR signaling, and a number of mechanisms have been proposed for reactivation of AR in the castrate environment (3,5). Novel endocrine treatments targeting the AR signaling axis, including abiraterone acetate and MDV3100, have recently shown clinical promise for advanced prostate cancer, particularly in the second-line therapeutic setting (6,7). Reports however suggest that resistance to these new agents, linked to continued hormone-driven oncogenesis, can develop (8). Thus the incomplete efficacy of androgen deprivation therapy highlights an urgent need for alternative treatment strategies to improve patient outcomes.

In this regard, targeting heat shock protein 90 (Hsp90) has emerged as a potential avenue for therapeutic intervention. Hsp90 is a molecular chaperone required for the post-translational stability and function of numerous key signal transduction proteins, termed ‘client’ proteins (9,10). Of note, a number of these clients have been causally implicated in the pathogenesis of prostate cancer, including AR, HER2, AKT and RAF1 (11–13). Interaction with Hsp90 regulates the half-life of these proteins and the AR is particularly reliant on Hsp90 function for its activity. Within the cytoplasm, the receptor is maintained in a multichaperone complex with Hsp90 that is essential for stabilizing the protein in a conformation receptive to ligand binding (14). Importantly, inhibition of Hsp90 activity targets its clients for proteasomal destruction. Thus pharmacological blockade of Hsp90 can overcome signaling redundancies and mechanisms of drug resistance commonly observed in many cancers (15–17) because of its coordinate and simultaneous impact on multiple signaling cascades. For these reasons, Hsp90 represents an attractive molecular target for the development of new anticancer agents (18,19).

A number of preclinical studies have provided compelling evidence supporting the potential utility of Hsp90 inhibitors in prostate cancer (20–23). Unfortunately, the clinical experience using such compounds in the single-agent setting has been disappointing, with minimal effects on PSA levels or tumor burden being observed along with unacceptable toxicities (24,25). Ganetespib (formerly STA-9090) is a new small molecule inhibitor of Hsp90 with superior pharmacologic and biologic properties that distinguish it from other first- and second-generation inhibitors in terms of antitumor activity, potency and safety (26). In light of these considerations, here we have undertaken a comprehensive evaluation of ganetespib activity in prostate cancer cell lines both *in vitro* and *in vivo*.

## Materials and methods

### Cell lines, antibodies and reagents

The LNCaP, VCaP, 22Rv1, DU145 and PC3 human prostate cancer cell lines and HeLa cells were all purchased from the American Type Culture Collection (Manassas, VA, USA). Cells were maintained and cultured according to standard techniques at 37°C in 5% (v/v) CO_2_ using culture medium recommended by the supplier. All primary antibodies were purchased from Cell Signaling Technology (Beverly, MA, USA) with the exception of RAF1 (Santa Cruz Biotechnology, Santa Cruz, CA, USA), p-EGFR (Tyr1068) (Invitrogen, Carlsbad, CA, USA), actin (GE Healthcare, UK) and the AR mouse monoclonal antibody AR441 (27), which was prepared by the antibody core of the Dan L. Duncan Cancer Center at Baylor College of Medicine. The Hsp90 inhibitors ganetespib and 17-AAG were synthesized at Synta Pharmaceuticals Corp. Methyltrienolone (R1881) was purchased from Perkin-Elmer (Boston, MA, USA).

### Cell viability assays

Cellular viability was assessed using the CellTiter-Glo Luminescent Cell Viability Assay (Promega, Madison, WI, USA) according to the manufacturer’s protocol. Twenty-four hours after plating at 5×10^3^ cells/well in triplicate in 96-well plates, cells were dosed with graded concentrations of ganetespib or 17-AAG for 72 h. CellTiter-Glo was added (50% v/v) to the cells, and the plates incubated for 10 min prior to luminescent detection in a SpectraMax Plus 384 microplate reader (Molecular Devices, Sunnyvale, CA, USA). Data were normalized to percent of control and IC_50_ values used to determine the sensitivity of each line.

### Western blotting

Prostate cancer cell lines were lysed in RIPA buffer (Cell Signaling Technology) and HeLa lysed by four rounds of freeze/thawing using 1X Reporter Lysis Buffer (Promega) containing 0.4 M NaCl. Lysates were clarified by centrifugation and equal amounts of protein resolved by SDS-PAGE before transfer to nitrocellulose membranes. Membranes were blocked with 5% skim milk in TBS with 0.5% Tween and immunoblotted with indicated antibodies. Antigen-antibody complexes were visualized using an Odyssey system (LI-COR, Lincoln, NE, USA) or using ECL reagents.

### Quantitative RT-PCR

LNCaP cells were cultured in charcoal-stripped medium for 24 h and then treated with 250 nM ganetespib, 1 *μ*M geldanamycin, or vehicle for 24 h in the absence or presence of 10 nM methyltrienolone (R1881). RNA was prepared from the LNCaP cells post-treatment using TRIzol reagent (Invitrogen, Grand Island, NY, USA). Previously reported prostate specific antigen (PSA), transmembrane protease, serine 2 (TMPRSS2), and 18S primer sets (28) were used for target gene expression and were analyzed using SYBR green PCR Master mix in an ABI 7500 Fast sequence detection system. PSA and TMPRSS2 mRNA levels were normalized to 18S mRNA values.

### Transient transfection of HeLa cells

HeLa cells were transiently transfected using a poly-L-lysine coupled adenoviral-mediated DNA transfer technique as previously described (29). The plasmid constructs used were pCR3.1-AR (encoding full-length AR) and pCR3.1-V7 (encoding the V7 truncated AR isoform, a gift from Manjula Nakka and William Krause, Baylor College of Medicine). For the expression study, HeLa cells were transfected with 3 ng of pCR3.1-AR or 0.5 ng of pCR3.1-V7 for 24 h. Cells were treated with R1881 (10 nM), GA (1 *μ*M), and/or ganetespib (250 nM) or vehicle (ethanol and DMSO) for 24 h prior to lysis and immunoblotting. To determine the effect of Hsp90 inhibitors on AR and variant activity, HeLa cells were transiently transfected with 250 ng of GRE-luciferase reporter, 30 ng of pCR3.1 β-galactosidase, 3 ng of pCR3.1-AR, or 0.03 ng of pCR3.1-V7 and treated as above except that inhibitors were added immediately after the completion of the transfection procedure. Luciferase and β-galactosidase activities were measured and luciferase levels normalized to β-galactosidase levels as previously described (30).

### Flow cytometry

For cell cycle analysis, PC3 and DU145 cells were seeded overnight at 0.3×10^6^ cells/5 ml in a 6-well plate and then exposed to increasing concentrations of ganetespib (0–500 nM) for 24 h. Cells were harvested and stained with propidium iodide using the BD Cycle Test Plus Reagent Kit (BD Biosciences, San Jose, CA, USA) according to the manufacturer’s instructions. Twenty thousand cells were analyzed for their DNA content using a FACS Calibur cytometer (BD Biosciences, Billerica, MA, USA). For the apoptosis assay in the DU145 cell line, cells were treated with ganetespib (10, 100 or 500 nM), 17-AAG (500 or 1000 nM) or control (DMSO) for 24 h. Following treatment cells were harvested and stained using a fluorescein-conjugated anti-Annexin V antibody (BD Biosciences) and apoptosis assessed by flow cytometry.

### In vivo prostate xenograft model

Eight-week-old female immunodeficient nude and CB-17 severe combined immunodeficient (SCID) mice (Charles River Laboratories, Wilmington, MA, USA) were maintained in a pathogen-free environment, and all *in vivo* procedures were approved by the Synta Pharmaceuticals Corp. Institutional Animal Care and Use Committee in accordance with the Guide for Care and Use of Laboratory Animals. PC3 tumor cells (5×10^6^) were subcutaneously implanted into nude mice and 22Rv1 cells (5×10^6^) into SCID mice. Animals bearing established tumors (100–200 mm^3^) were randomized into treatment groups of 8 and i.v. dosed via the tail vein with either vehicle or ganetespib formulated in 10/18 DRD (10% DMSO, 18% Cremophor RH 40, 3.6% dextrose, 68.4% water). Tumor volumes (V) were calculated by caliper measurements of the width (W), length (L), and thickness (T) of each tumor using the formula: V = 0.5236 (LWT). Tumor growth inhibition was determined as described previously (31).

## Results

### Ganetespib potently induces cell death in prostate cancer cells irrespective of androgen receptor status

We initially examined the growth inhibitory effects of ganetespib *in vitro* using a panel of prostate cancer cell lines. In all cases, ganetespib reduced cell viability in a dose-dependent manner and was more potent than the first-generation ansamycin Hsp90 inhibitor 17-AAG (Table I). In the AR-negative cell lines DU145 and PC3 the cytotoxicity IC_50_ values at 72 h were 12 and 77 nM, respectively. The AR-positive, androgen-dependent cell lines LNCaP and VCaP were more sensitive to ganetespib exposure (IC_50_ values of 8 and 7 nM). The 22Rv1 cell line, which while AR-positive is only weakly androgen responsive, was also highly sensitive to ganetespib (IC_50_, 20 nM). These data demonstrate that Hsp90 inhibition by ganetespib results in potent cytotoxic effects in prostate cancer lines regardless of their AR status or androgen sensitivity.

### Coordinate inhibition of AR activity and multiple oncogenic signaling pathways in prostate cancer cells by ganetespib

**Figure 1.**
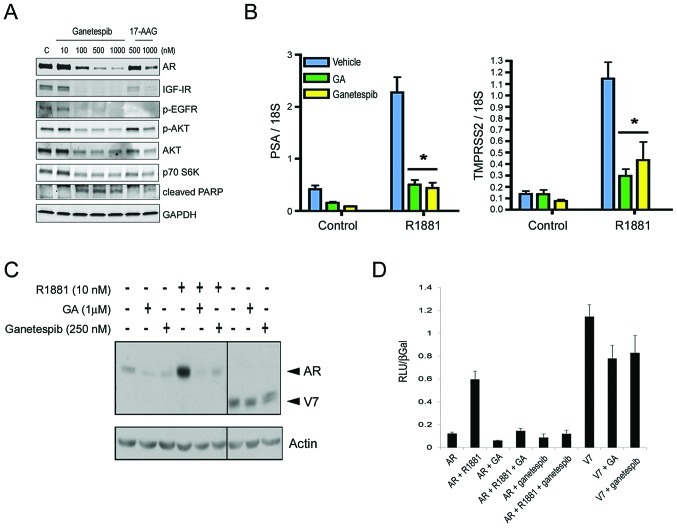
Ganetespib treatment destabilizes full-length AR receptor expression and activity, as well as multiple client proteins, in AR-positive cancer cell lines. (A), LNCaP cells were exposed to increasing concentrations of ganetespib or 17-AAG as indicated for 24 h. Cell lysates were immunoblotted using antibodies against AR, IGF-IR, phosphorylated EGFR (p-EGFR), phosphorylated AKT (p-AKT), total AKT and p70 S6K as shown. Cleaved PARP expression was included as a marker of apoptosis. Total protein levels were determined using GAPDH. (B), LNCaP cells were cultured in charcoal-stripped medium for 24 h and then treated with 250 nM ganetespib, 1 *μ*M geldanamycin (GA), or vehicle for 24 h in the absence or presence of 10 nM androgen (R1881). Prostate specific antigen (PSA) and transmembrane protease, serine 2 (TMPRSS2) mRNA levels were measured and normalized to 18S mRNA values. Experiments were performed in triplicate. Androgen-inducible transcriptional activation was significantly inhibited in the presence of either Hsp90 inhibitor (^*^P<0.001). (C), HeLa cells were transiently transfected with 3 ng of pCR3.1-AR or 0.5 ng of pCR3.1-ARV7 plasmid to induce expression of the full-length and V7 truncated AR proteins, respectively (arrowheads). Twenty-four hours following infection, cells were treated with 10 nM R1881, 1 *μ*M GA, or 250 nM ganetespib as indicated. Cell lysates were resolved by SDS-PAGE and immunoblotted with an anti-AR antibody. Total protein levels were determined using an anti-actin antibody. (D), To determine the effect of Hsp90 inhibitors on AR and variant activity, HeLa cells were transiently transfected with 250 ng of GRE-luciferase reporter, 30 ng of pCR3.1 β-galactosidase, 3 ng of pCR3.1-AR, or 0.03 ng of pCR3.1-V7 and treated with vehicle (ethanol and DMSO), R1881 (10 nM), GA (1 *μ*M), and/or ganetespib (250 nM) for 24 h. Luciferase and β-galactosidase activities were measured and luciferase levels were normalized to β-galactosidase levels. Experiments were performed in triplicate.

Targeted degradation of client proteins is a feature of Hsp90 inhibition. We therefore examined expression changes in Hsp90 clients known to be associated with prostate tumor progression. AR-positive LNCaP cells were treated with ganetespib or 17-AAG for 24 h and protein levels determined by western blot analysis (Fig. 1A). Ganetespib treatment resulted in a potent and dose-dependent decrease in AR levels. Hsp90-directed loss of AR receptor expression resulted in consequent suppression of AR-directed gene regulation. To show this, LNCaP cells were cultured in charcoal-stripped medium for 24 h and then treated with ganetespib, geldanamycin (GA, the parent compound from which 17-AAG is derived), or vehicle for 24 h in the absence or presence of androgen (R1881). As a read-out of AR-specific transcriptional activity, PSA and TMPRSS2 mRNA levels were measured and normalized to 18S mRNA values (Fig. 1B). In accordance with the androgen-inducible expression of both genes, R1881 exposure increased PSA and TMPRSS2 levels in control cells. This induction was significantly inhibited in the presence of either Hsp90 inhibitor (^*^P<0.001) (Fig. 1B).

Importantly, ganetespib also induced degradation of IGF-IR and phosphorylated EGFR receptors, previously implicated in the pathogenesis of prostate cancer, as well as the downstream effectors AKT and p70 S6K, in LNCaP cells (Fig. 1A). Moreover a concomitant increase in PARP cleavage, a marker of apoptosis, accompanied the reductions in these protein levels. Consistent with the differences in sensitivity shown in Table I, ganetespib was comparatively more potent than 17-AAG at inducing targeted loss of these oncogenic proteins and signaling pathways.

### Constitutively active AR variant expression does not confer resistance to ganetespib

The expression of alternatively spliced, terminally-truncated AR isoforms is one potential mechanism for the development of a castration-resistant phenotype (32). For example, the 22Rv1 cell line expresses the full-length AR protein as well as constitutively active variants that lack the carboxyl-terminal ligand-binding domain (33,34), thereby reducing its dependence on exogenous androgen. Of note, we found that 22Rv1 cells were acutely sensitive to the effects of ganetespib treatment (Table I), although loss of the truncated receptor appeared less pronounced than that of full-length AR following treatment (data not shown). To directly examine the effects of Hsp90 inhibition on alternate receptor proteins, we transiently transfected plasmids encoding full-length AR as well as the truncated isoform corresponding to the known V7 variant (33) into HeLa cells (Fig. 1C). Androgen treatment increased full-length AR expression at 24 h and this response was completely abrogated in the presence of either 1 *μ*M GA or 250 nM ganetespib. Both Hsp90 inhibitors were also effective at targeted degradation of AR in the absence of androgen stimulation; however neither inhibitor significantly altered expression of the variant receptor (Fig. 1C). Similarly, GA and ganetespib strongly inhibited full-length AR activity but were less effective against constitutive V7 activity measured using an AR responsive luciferase reporter assay (Fig. 1D). Although the truncated V7 isoform appears less sensitive to Hsp90 inhibition, the potent activity of ganetespib in 22Rv1 cells suggests that its concomitant impacts on multiple signaling pathways can overcome any potential selective advantages provided by constitutively active variant expression.

### Ganetespib inhibits multiple oncogenic Hsp90 client proteins in AR-negative prostate cancer cells to induce cell death

**Figure 2.**
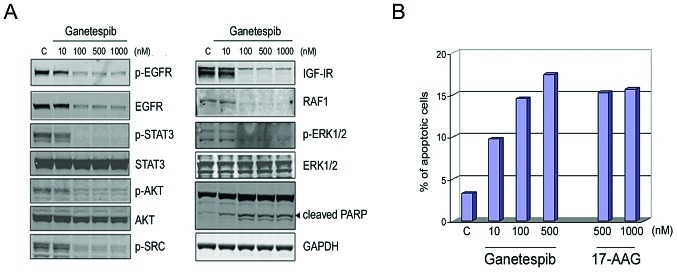
Ganetespib inhibits multiple Hsp90-dependent signaling pathways in AR-negative DU145 prostate cells to induce apoptosis. (A), DU145 cells were exposed to graded concentrations of ganetespib as indicated for 24 h. Cell lysates were immunoblotted using antibodies against phosphorylated EGFR (p-EGFR), total EGFR, phosphorylated STAT3 (p-STAT3), total STAT3, phosphorylated AKT (p-AKT), total AKT, phosphorylated SRC (p-SRC), IGF-IR, RAF1, phosphorylated ERK1/2 (p-ERK1/2) and total ERK1/2 as shown. Cleaved PARP expression is included as a marker of apoptosis. Total protein levels were determined using GAPDH. (B), DU145 cells were treated with ganetespib (10, 100 or 500 nM), 17-AAG (500 or 1000 nM) or control (DMSO) for 24 h. Cells were harvested, stained with a fluorescent conjugated anti-Annexin V antibody and apoptosis measured by flow cytometry.

The DU145 prostate cancer cell line lacks AR receptor expression. However, the growth and survival of these cells has been reported to be regulated through autocrine activation of EGFR by its ligands (35), in turn leading to oncogenic STAT activation. Further, this line also expresses an autocrine IL-6 cytokine signaling loop that results in persistent activation of the JAK/STAT signaling pathway (36). Ganetespib effectively targeted EGFR and completely abrogated STAT3 signaling in these cells in a dose-dependent manner (Fig. 2A). In addition, IGF-IR and downstream signaling pathways mediated through p-AKT, RAF1, and p-ERK1/2 were also destabilized following ganetespib exposure, similar to that observed in LNCaP cells (Fig. 1A). The correlative increase in cleaved PARP expression indicated that simultaneous blockade of these signaling pathways triggered apoptosis and this was further supported by Annexin V staining (Fig. 2B). Cells were treated with escalating doses of ganetespib or 17-AAG for 24 h and then analyzed by flow cytometry. Ganetespib treatment resulted in a dose-dependent increase in apoptotic cells. A comparable proportion of apoptotic cells was seen following high doses of 17-AAG, a response that was saturated by the 500 nM exposure level.

### Kinetics of Hsp90 client protein degradation by ganetespib

**Figure 3.**
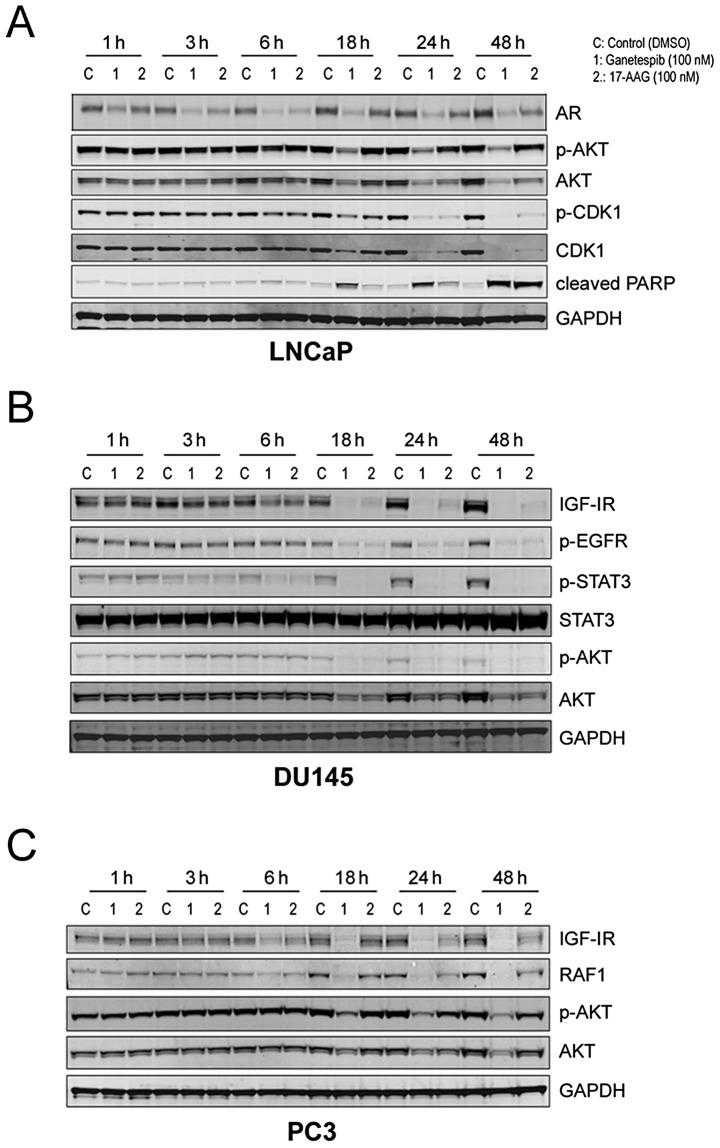
Comparative kinetics and potency of Hsp90 client protein destabilization by ganetespib and 17-AAG in prostate cell lines. Effect of Hsp90 inhibition by: 1, ganetespib or 2, 17-AAG in LNCaP (A), DU145 (B) or PC3 (C) cells was assessed. Cell lines were exposed to 100 nM concentrations of either inhibitor and harvested at 1, 3, 6, 18, 24 and 48-h post-treatment. Cell lysates were resolved by SDS-PAGE and immunoblotted with the indicated antibodies.

We next examined the kinetics of client protein loss in response to Hsp90 inhibition. In LNCaP cells, 100 nM ganetespib treatment rapidly (within 3 h) resulted in a measurable reduction in AR expression and this effect was sustained over a 48-h time course (Fig. 3A). Destabilization of p-AKT/AKT was a relatively later event occurring at 18 h; these kinetics matched those observed for the elevation of cleaved PARP. Interestingly, ganetespib also induced a loss of both the total and phosphorylated forms of cyclin dependent kinase 1 (CDK1), a key regulator of the G_2_/M checkpoint, by 24 h and this effect persisted until at least 48 h (Fig. 3A).

The kinetics of targeted AKT degradation were similar in the AR-negative prostate cell lines DU145 and PC3 (Fig. 3B and C, respectively). In DU145 cells, significant reductions in p-EGFR expression also required an 18-h exposure to either ganetespib or 17-AAG, whereas destabilization of IGF-IR and p-STAT3 was evident by 6 h (Fig. 3B). Like LNCaP cells, PC3 prostate cells were significantly more sensitive to the effects of ganetespib treatment compared to an equivalent dose of 17-AAG (Fig. 3C). Consistent with the DU145 results, ganetespib reduced IGF-1R levels in this line by 6 h and sustained loss of the receptor was observed over the 48-h time course. In addition, a potent and time-dependent reduction in RAF1 protein expression which also preceded AKT modulation was observed (Fig. 3C).

### Modulation of cell cycle protein expression by ganetespib induces growth arrest and apoptosis

**Figure 4.**
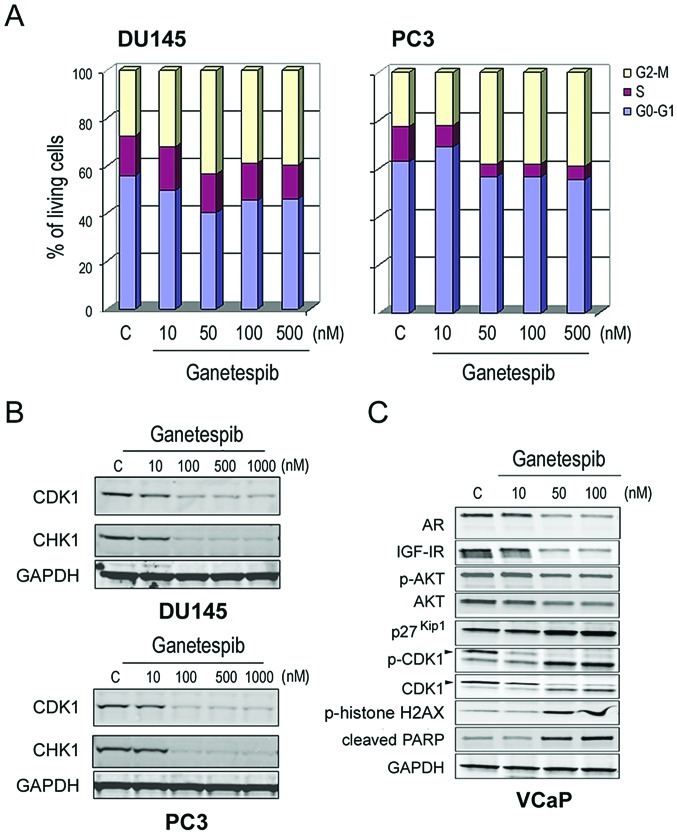
Ganetespib modulates cell cycle protein expression and induces growth arrest in prostate cancer cells. (A), DU145 and PC3 cells were treated with increasing concentrations of ganetespib as indicated. Cell cycle distribution was determined in each line by flow cytometry 24-h post-treatment. (B), DU145 and PC3 cells were treated with increasing concentrations of ganetespib for 24 h as in (A). Cell lysates were immunoblotted using antibodies against CDK1, CHK1 and GAPDH. (C), VCaP cells were treated with ganetespib at 0, 10, 50 and 100 nM for 24 h. Cell lysates were immunoblotted using antibodies against AR, IGF-IR, phosphorylated AKT (p-AKT), total AKT, p27^Kip1^, phosphorylated-CDK1 (p-CDK1), total CDK1, phosphorylated (p-)histone H2AX, PARP and GAPDH.

We have previously reported that ganetespib treatment can exert profound effects on cell cycle regulatory proteins, in addition to oncogenic signaling pathways, that contribute to its antitumor activity (31). Cell cycle analysis revealed that ganetespib exposure led to a dose-dependent accumulation of cells in the G_2_/M phase in both DU145 and PC3 cells, with a concomitant loss of S phase (Fig. 4A). In both cell lines, we observed a corresponding reduction in protein expression of CDK1 as well as CHK1, another kinase that plays an essential role in the integrity of the G_2_/M checkpoint (Fig. 4B). Next, more extensive characterization of the concomitant impact of ganetespib on both oncogenic and cell cycle signaling was performed in androgen-dependent VCaP prostate cells. As seen in the LNCaP line (Fig. 1A), ganetespib treatment of these cells induced AR and IGF-IR degradation and reduced p-AKT/AKT levels in a dose-dependent manner (Fig. 4C). In agreement with recent findings (37), ablation of AR/AKT signaling resulted in accumulation of the cyclin-dependent kinase inhibitor p27^Kip1^. In addition, loss of both the total and phosphorylated forms of CDK1 was observed as a function of dose. Taken together, these data suggest that loss of checkpoint control and G_2_/M arrest accompanies blockade of oncogenic signaling in prostate cancer cells as a result of Hsp90 inhibition by ganetespib. Moreover, we observed concomitant elevations in phosphorylated histone H2AX and PARP cleavage (Fig. 4C). Since the phosphorylated form of H2AX is a sensitive indicator of DNA double strand break formation, these data suggest that G_2_/M arrest leads to subsequent apoptosis.

### Ganetespib inhibits AR-dependent and AR-independent tumor growth in vivo

**Figure 5.**
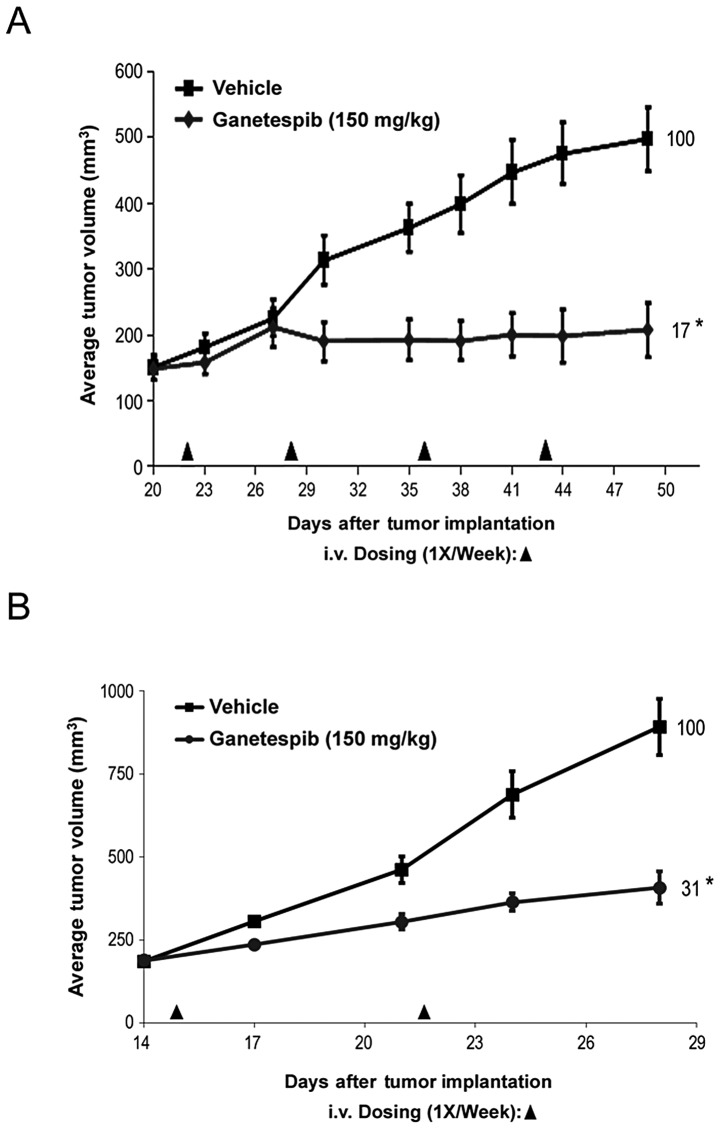
Suppression of *in vivo* prostate tumor growth by ganetespib. (A), Nude mice bearing established PC3 prostate xenografts were i.v. dosed with ganetespib (150 mg/kg) or vehicle (n=8 mice/group) on a weekly dosing schedule as indicated (arrowheads) for 4 weeks. (B), SCID mice bearing established 22Rv1 xenografts were i.v. dosed with ganetespib (150 mg/kg) or vehicle (n=8 mice/group) on a weekly dosing schedule as indicated (arrowheads). Tumor volumes were measured by caliper. Results are presented as mean ± SEM. The reduction in tumor volume in ganetespib-treated animals for both studies was significant (^*^P<0.05, ANOVA).

Finally, to determine whether the potent *in vitro* effects of ganetespib translated to *in vivo* antitumor activity, we studied the efficacy of single-agent ganetespib treatment on the growth of prostate cancer xenografts. We have previously determined that the highest non-severely toxic dose of ganetespib on a weekly dosing regimen is 150 mg/kg (26). As shown in Fig. 5A, mice bearing AR-independent PC3 xenograft tumors treated on this schedule exhibited a significant decrease in tumor volume compared to control animals (T/C value 17%). Even at this dose the regimen was well tolerated. Minor body weight losses occurred post-administration but were rapidly recovered between dosing points and no net loss of body weight was observed over the duration of the study (data not shown). Ganetespib treatment was also highly efficacious in rapidly growing xenografts derived from the AR-dependent 22Rv1 cell line (Fig. 5B), consistent with the sensitivity data obtained *in vitro*. Together, these data show that ganetespib treatment can significantly inhibit prostate tumor growth, again irrespective of androgen receptor status.

## Discussion

Androgen ablation therapy has been a mainstay of prostate cancer treatment since the concept was first introduced over 70 years ago (38). For advanced disease, however, this approach has not proven curative since durable tumor suppression is typically not achieved and patients invariably progress to a castrate-resistant phenotype. Until the introduction of docetaxel as a standard of care in 2010, first-line chemotherapeutics such as estramustine and mitoxantrone failed to provide overall survival benefit to patients with advanced or recurrent tumors (39). More recently, an improved understanding of the underlying biology of prostate cancer has led to major clinical and translational advances, particularly in the development of novel androgen-ablative and AR antagonist strategies (6,7). Even with such progress, an urgent need for more effective and alternative approaches to combat the disease remains. In this era of molecularly targeted therapies Hsp90 inhibition has emerged as an exciting potential avenue of therapeutic intervention in a variety of human malignancies, including prostate tumors (15,40). To date, however, the clinical experience with first-generation ansamycin inhibitors of Hsp90 in prostate cancer has been disappointing, hampered by poor single-agent activity and adverse toxicity profiles (24,25).

Ganetespib is a unique resorcinolic triazolone small molecule inhibitor of Hsp90, structurally unrelated to the ansamycin class, which exhibits potent activity in a broad range of preclinical models of human malignancies (26,41). Moreover, ganetespib displays superior pharmacological and safety properties compared to other Hsp90 inhibitors and is currently undergoing clinical evaluation in multiple phase I and II trials. Here we examined the effects of ganetespib in a panel of prostate tumor lines and in both AR-dependent and independent xenograft models. With low nanomolar potency, ganetespib reduced cell viability *in vitro* in all the lines examined, irrespective of their androgen sensitivity and/or AR status. Ganetespib exposure resulted in a dose-dependent destabilization of multiple Hsp90 client proteins, including AR, EGFR, IGF-IR and AKT. Importantly, ganetespib demonstrated superior potency and more durable responses in terms of client protein suppression compared to 17-AAG. Thus, while ganetespib exerts its pharmacological effects on Hsp90, it was clear that the downstream consequences involved an array of client proteins and biochemical pathways. The combinatorial blockade of multiple key signaling components required for prostate cancer cell growth and survival, and subsequent induction of apoptosis, accounted for the potent cytotoxic activity of the compound.

The AR is an established Hsp90 client, and the relationship between the chaperoning function of Hsp90 with steroid receptor stability, conformation and modulation of ligand binding is well characterized (reviewed in ref. 42). For the AR-expressing cell lines, abrogation of this critical signaling axis by ganetespib likely underlies their acute sensitivity. For example, and in agreement with studies of other Hsp90 inhibitors (21,23), ganetespib treatment of LNCaP cells promoted the rapid degradation of AR expression. This was accompanied by inhibition of AR-transactivation and AR-dependent gene expression. Of note, destabilization of AR was an early event, and preceded the downstream loss of AKT signaling and induction of apoptosis by several hours, highlighting the importance of Hsp90 for stability and function of the steroid receptor.

In AR-negative cell lines ganetespib exposure resulted in the simultaneous disruption of signaling networks that have been implicated in the aberrant growth and survival of prostate cancer. IGF-IR activation has mitogenic and antiapoptotic effects in prostate tumor cells and circulating serum levels of IGF1 have been associated with increased risk of prostate cancer (43,44). Ganetespib treatment of DU145 and PC3 cells led to the potent and sustained degradation of IGF-IR and its downstream effector pathways PI3K/AKT and ERK1/2. In addition persistent STAT3 activation, through either autocrine cytokine or EGFR activation, is a feature of DU145 cells (35,36) and this pathway was also effectively inhibited following ganetespib exposure. Thus, targeting Hsp90 can overcome the compensatory signaling pathways present in androgen-insensitive prostate cancer cells that promote aberrant cell survival. Taken together, these data suggest that ganetespib may be effective in controlling castrate-resistant disease. In support of this premise, we observed encouraging antitumor efficacy of ganetespib as a single agent in an *in vivo* study using the PC3 xenograft model. Additional efficacy and pharmacodynamic studies of ganetespib in preclinical models of androgen-dependent and castrate-resistant prostate cancer are underway.

It has been proposed that one mechanism that may contribute to the development of a castration resistant phenotype is the expression of truncated, constitutively active AR isoforms, including the well described V7 variant (32–34). This variant, expressed by the 22Rv1 cell line, has been shown to be enriched in xenograft models of androgen-refractory prostate cancer, to promote the growth of androgen-dependent xenografts in castrate mice, and to be upregulated in malignant human prostate tissues compared to their benign counterparts (32,45). Interestingly, we found that while full-length AR was potently destabilized following Hsp90 blockade, ganetespib had minimal effect on V7, either in terms of its targeted degradation or inhibition of its constitutive transactivation activity. It has been well established that AR associates with Hsp90, through its ligand binding domain, in order to adopt a confirmation that is competent to bind ligand. In the case of the constitutively active V7, which lacks the ligand binding domain, this interaction is likely no longer required; a property distinct to most other client proteins that are generally more reliant on Hsp90 for their stability and function (10,16). Despite this apparent lack of direct Hsp90 modulation, the acute sensitivity of the 22Rv1 line *in vitro* as well as the antitumor efficacy observed *in vivo* indicates that ganetespibs’ multifaceted mode of action can bypass the selective advantages provided by truncated AR isoform expression.

We have previously shown that the cellular impact of Hsp90 inhibition by ganetespib is not restricted to oncogenic survival signaling but also includes profound effects on the cell cycle regulatory machinery (31). In the data presented here, ganetespib exposure resulted in G_2_/M accumulation and loss of S phase in prostate cancer cells, mediated at least in part through loss of the checkpoint regulatory proteins CDK1 and CHK1. In this regard, it is known that Hsp90 inhibition can sensitize cancer cells to the effects of chemotherapy and radiotherapy (46,47), and modulation of the cell division machinery represents an important component of this cytotoxic sensitizing property. For example, depletion of CHK1 and loss of checkpoint control as a result of Hsp90 inhibition has been reported to enhance the cytotoxic activity of the chemotherapeutic agents gemcitabine and irinotecan (48,49). Moreover, we recently showed that ganetespib synergistically potentiated the cytotoxic effects of taxanes, a group of microtubule-targeted agents that cause mitotic catastrophe, in preclinical models of non-small cell lung cancer (41). Interestingly, mitotic disruption can also be exacerbated by Hsp90 inhibition in cell lines with defects in the function of the retinoblastoma (RB) tumor suppressor protein (50), presumably linked to interference with Hsp90’s role in centrosome organization (51,52). RB is a master cell cycle regulator and key component of the proliferative response to AR which is lost or inactivated with high frequency (30–60%) in prostatic neoplasms (2). Taken together, these findings provide additional evidence for the potential advantages of Hsp90 inhibitors such as ganetespib, based on their multifaceted mode of action, to overcome deficiencies of AR-directed therapeutics.

In conclusion, we have shown that the unique small molecule Hsp90 inhibitor ganetespib exhibits robust cytotoxic activity and antitumor efficacy in preclinical models of prostate cancer. Importantly, due to concomitant effects on oncogenic survival pathways and cell cycle progression, ganetespib treatment potently induced cancer cell death irrespective of androgen sensitivity. Together, the data suggest that ganetespib may serve as an effective treatment strategy for prostate cancers driven by AR, truncated forms of the receptor that confer androgen independence, as well as castrate-resistant tumors no longer reliant on the receptor itself. In light of these findings, further evaluation of the therapeutic utility of this agent is warranted.

## Figures and Tables

**Table I.** Comparison of ganetespib and 17-AAG *in vitro* cytotoxicity in a panel of prostate cancer cell lines.

Cell line	AR expression/androgen sensitivity	Ganetespib (nM)	17-AAG (nM)
LNCaP	+/Dependent	8	266
VCaP	+/Dependent	7	2645
22Rv1	+/Partial	20	1270
DU145	−/Independent	12	36
PC3	−/Independent	77	246
